# Maternal vaccine delivery costs in South Asian settings: estimates from Bangladesh and Nepal

**DOI:** 10.1186/s12889-025-25786-3

**Published:** 2025-12-03

**Authors:** Ranju Baral, Jessica A. Fleming, Abhiyan Gautam, Md. Monjurul Islam, Gauri Shrestha, Sadaf Khan, Mohammed M. Kashem, Sandeep Kumar, Bibek Lal, Kamran Mehedi, Iffat R. Nasir, Lauren Newhouse, Satyabrata Routray, Jayanto K. Saha, Sarah Sultana, Surendra Uranw, Clint Pecenka

**Affiliations:** 1 Center for Vaccine Innovation and Access, PATH, 437 N 34th Street, Seattle, WA 98103 USA; 2 Ministry of Health, Kathmandu, Nepal; 3 Ministry of Health, Dhaka, Bangladesh; 4 PATH, Dhaka, Bangladesh; 5 PATH, New Delhi, India; 6 Independent consultant, Dhaka, Bangladesh; 7 BP Koirala Institute of Health Sciences (BPKIHS), Dharan, Nepal

**Keywords:** Maternal vaccines, Cost, Cost of delivery, Financial cost, Economic cost

## Abstract

**Background:**

Several maternal vaccines are in clinical development. Maternal respiratory syncytial virus (RSV) vaccine has been introduced in several high and upper middle-income countries. With the recent World Health Organization (WHO) RSV prevention recommendation and Gavi, the Vaccine Alliance’s commitment to open a funding window for RSV maternal vaccine programs, the vaccine will soon be available for low- and middle-income markets. Understanding costs of implementing new maternal vaccines within existing health systems in low-and middle-income countries (LMICs) is critical to inform introduction decisions. This study projects the cost of maternal immunization (MI) introduction and delivery in Bangladesh and Nepal.

**Methods:**

Using an activity based prospective costing approach, we project MI introduction and delivery costs for a five-year period in each country. In-country stakeholders informed the future MI delivery strategies used for costing. Interviews with immunization and maternal health program representatives informed activities and resource needs. Primary data from a sample of sub-national health administrative units including vaccine stores and health facilities also informed anticipated MI operational costs. Financial and economic costs to the health system are estimated and reported in 2023 US$ units.

**Results:**

Stakeholders confirmed utilizing existing maternal tetanus vaccine delivery systems as the most feasible strategy for future MI delivery in both countries. The non-vaccine cost of delivering one dose of maternal vaccine was estimated at $0.45 and $1.81 (financial) and $2.01 and $4.49 (economic) in Bangladesh and Nepal, respectively. Excluding commodity cost, health worker training and demand generation activities were among the largest cost drivers in both countries. Relatively lower unit costs in Bangladesh are partly due to larger target population size leading to some efficiency in introduction costs. The anticipated coverage and baseline health system capacity gaps also contribute to the variation in unit costs between countries.

**Conclusions:**

MI delivery costs in LMICs are little known and this study contributes to filling this gap. These cost projections can equip countries to make informed introduction decisions as they evaluate the affordability and sustainability of MI programs in respective countries.

**Supplementary Information:**

The online version contains supplementary material available at 10.1186/s12889-025-25786-3.

## Background

Vaccination of pregnant populations to protect them and their newborns, often referred to as maternal immunization (MI), is an emerging tool in public health to curb infectious diseases [1]. A new maternal respiratory syncytial virus (RSV) vaccine was first approved by the US and Europe [2, 3] and rollouts are cascading, mostly in high-and upper-middle-income countries [4]. Several other maternal vaccine candidates are currently under development, including advanced candidates for diseases like Group B *Streptococcus* (GBS) [5, 6]. Recently, the World Health Organization’s Strategic Advisory Group of Experts on Immunization (SAGE) recommended broader use of RSV maternal vaccine [7] and, subsequently, the multilateral vaccination funder, Gavi, the Vaccine Alliance approved support for this vaccine [8], contributing to accelerated access to these products in low-and middle-income countries (LMICs).

Over the last several decades, tetanus toxoid containing vaccines (TTCV) have been delivered to millions of pregnant women across the globe, contributing to maternal and neonatal tetanus elimination in many countries [9]. Currently available maternal vaccines (including TTCV) are often delivered by leveraging parts of the antenatal care (ANC) platform. Some new maternal vaccines and target diseases bring unique features that may require adaptations to existing maternal vaccine delivery mechanisms. For example, countries may consider seasonal delivery of maternal RSV vaccines to coincide with the circulation of RSV. Vaccines may also be recommended to be delivered during a restricted gestation age window [2, 10]. As additional maternal vaccines become available for use in LMICs existing health systems may require adaptations to optimize delivery, specifically within the immunization and maternal health care programs [11, 12]. Adaptation of the health systems to create effective and efficient maternal vaccine delivery platforms have cost consequences.

Understanding the costs associated with new vaccine introduction and delivery is an important component of country introduction decisions and priority setting. While the literature on costs of immunization programs across the globe is expanding [13], information on the costs of delivering vaccines in pregnancy is limited, especially for LMICs [14]. Understanding cost of delivery (COD) and introduction implications for MI is important for informing policy decisions.

To address gaps in MI delivery costs, we conducted a costing study across five countries in Asia and Africa to generate empirical evidence on the potential costs of delivering maternal vaccines as part of a MI platform. In this paper, we present findings from two south Asian countries: Bangladesh and Nepal. Findings from the other countries are discussed elsewhere [15, 16]. The COD estimates generated by this study can inform the respective country as well as global decision-making on the economic feasibility of implementing new maternal vaccines. Findings from this study can also contribute to broader analyses of the cost-effectiveness and affordability of integrating new MI interventions into public health systems.

## Methods

In Bangladesh and Nepal, existing maternal vaccines provided in pregnancy are typically delivered by the Expanded Programme on Immunization (EPI), leveraging parts of the ANC delivery platform. Pregnant women attending ANC clinics are referred to the EPI to receive vaccination. As more maternal vaccines become available in the future, efficient and effective delivery of such interventions may necessitate adaptations to the existing MI delivery platforms [12].

To understand the country-specific needs for strengthening the MI platform based on the capacities and gaps within the immunization and ANC platforms, the study team first hosted in‑country stakeholder workshops to deliberate on the feasible strategies for MI delivery. The workshops included 57 (Bangladesh) and 37 (Nepal) participants, including policy decision-makers at national and regional levels, program practitioners from the ministries of health (MOH), academics, researchers, and development partner representatives. Workshop participants concluded that each country’s existing service delivery platforms were best positioned to deliver new maternal vaccines, though key areas would need strengthening in advance of introduction. Findings from workshops are discussed elsewhere [17].

### Study scope

Guided by insights from the stakeholder workshop, we generated MI introduction and delivery cost projections from the perspective of the country health system. We used a five-year timeframe for cost projections and assumed nationwide vaccine introduction starting in 2024. We selected 2024 to ground cost estimates at a timepoint in the near future, although new maternal vaccines may not be available in these settings for several years [4]. The analysis considered all pregnant women eligible to receive a single dose of maternal vaccine between 24 and 36 weeks of pregnancy, as per the RSV maternal vaccine schedule in the target product profile [4, 18]. As informed by the stakeholder workshops, vaccine delivery was assumed to be delivered in the same manner as TTCV delivery in each country. Adaptations needed for creating a conducive MI delivery platform were considered as a part of the costing. Only costs incremental to the existing delivery platform were considered. Other than the assumption that the vaccine doses would be donated to countries, the analysis did not include other potential donor support for MI introduction nor vaccine financing mechanisms.

### Costing approach

We used an activity-based costing method to identify and individually cost necessary activities for implementing MI using a mapping conducted with key MOH focal points from immunization and maternal and child health programs. All levels of the health system were included. Activities included those associated with the introduction of most vaccines, such as program planning and management, vaccine procurement, vaccine distribution and cold chain maintenance, training, communication and demand generation, and monitoring and supervision. For each activity category, sub-activities were identified reflecting current capacities for existing maternal vaccine delivery and adaptation needed for future MI integration and delivery (Appendix Table 1).

The final activity list was mapped to corresponding resource requirements. By activity, quantities of each resource requirement (i.e., labor, allowances, supplies) were estimated and multiplied by the respective unit cost to generate activity-specific cost estimates. Costing analyses followed the standard guidelines in estimating the potential costs of new vaccine introduction and delivery through the existing or an adapted health service delivery platform [19–22].

Costed activities were categorized into introduction and recurrent costs. Introduction costs included costs associated with initial set-up activities such as introduction planning, training, social mobilization, and capital resource purchases like cold chain equipment. Costs of introductory activities were considered as capital costs and, therefore, annualized and discounted at 3% over a 5-year useful life for baseline estimates. Recurrent costs included costs of routine program operations such as procurement and distribution of vaccine supplies, vaccine delivery, and monitoring and supervision, etc. Costs of capital item purchases, including cold chain equipment, were annualized over their respective estimated useful life years.

Cost estimates are presented as financial and economic costs. Financial cost represents direct monetary outlays or value of resources that are purchased by payers such as outreach allowances and per diems, as well as resources used in training and developing new communication materials. These exclude resources already paid for or owned by the government such as salaries of existing health workers. Economic costs, on the other hand, include all financial costs plus the value of in-kind resources used for interventions (e.g., salaries of current health personnel, volunteer labor, donated supplies), and the opportunity cost of capital goods, where applicable.

Vaccine doses were assumed to be donated by development partners; therefore, vaccine cost was excluded from the financial cost estimates of vaccine procurement but included in economic vaccine procurement cost estimates. Cost of immunization supplies (such as syringes and safety boxes) and the procurement add-on costs (freight and shipping/handling costs) on vaccine doses and immunization supplies are included as financial costs to the payer. In practice, countries are anticipated to contribute to vaccine procurement costs, partially subsidized by donor funding (e.g., Gavi). Similarly, donor funding may fully or partially cover injection supplies and associated logistics including freight and handling. Given the uncertainties around what and how much is included in the cost share related to vaccine commodities, cost estimates are presented separately for commodity procurement and non-commodity delivery costs to facilitate interpretation and comparison.

### Data collection

We collected data to inform cost projections in the following ways: (1) key informant interviews, (2) health facility surveys, and (3) secondary data (administrative data and financial records) review. The survey tools are available in the supplementary file. We conducted key informant interviews with the EPI and maternal and child health program leads and relevant focal points, who were purposively selected based on their roles in the immunization and maternal and child health care fields. Informants were primarily from the national level, with relevant representation from sub-national health administrative units. Surveys at health facilities and vaccine stores informed potential costs of MI delivery and provided contextual insights to inform costing. A sample of health administrative units, vaccine stores, and health facilities were purposively selected in both countries with inputs from the national programs to capture variation in geography, health care access, and health system performance across each country. Sample sizes differed between the two countries. In Bangladesh, data came from the following administrative divisions (*N* = 4): Dhaka, Chattogram, Rangpur, and Sylhet. We selected one district in each division (*N* = 4) and included two upazilas (or sub-districts) from each district (*N* = 8). We interviewed heads of three health facilities in each upazila (*N* = 24) as well as the respective upazila (*N* = 8) and district vaccine store in-charges (*N* = 4). In Nepal, we interviewed individuals from the national level, two provinces (*N* = 2), and three districts (*N* = 3) within the selected provinces (two from Province 1 [Koshi Province] and one from Province 2 [Madesh Province]). We also interviewed five heads of health facilities (*N* = 5) and the respective vaccine stores (*N* = 6). List of sample areas and facilities surveyed for costing is included in Appendix Table 2.

Secondary data from administrative databases informed inputs such as target population and expected vaccination coverage. Vaccine quantities and supplies were estimated using the size of key service recipient populations (i.e., pregnant women). Maternal vaccine coverage was estimated from the coverage of four or more ANC visits (ANC4+) by province in Nepal and two or more doses of TTCV coverage in Bangladesh (Table 1). We estimated input costs by reviewing the financial expense records from previous years. Additionally, we used recent new vaccine introduction planning documents to supplement information on unit costs and resource requirements.

**Table 1:** Key data inputs and assumptions used for cost projections

Inputs	Bangladesh	Nepal	Data sources
Target population and coverage			
Target population, number of pregnant women	4,007,544	515,533	**Bangladesh**: District Health Information System (DHIS) 2 report, 2023 **Nepal**: Expanded Programme on Immunization (EPI) 2023, Central Bureau of Statistics, Nepal
Population growth rate	1.5%	0.9%	**Bangladesh**: Assumed **Nepal**: EPI 2023, Central Bureau of Statistics, Nepal
Vaccination coverage (range across region)	90%(range not available)	75%(49%–100%)	**Bangladesh**: Tetanus-diphtheria two-dose (TD2+) vaccine coverage, Coverage Evaluation Survey report, 2019 **Nepal:** ANC4+ coverage, EPI 2022
Product characteristics			
Presentation	3 doses per 2 ml vial, lyophilized		Assumption
Diluent	3 doses per 2 ml vial		
Vaccine packaged volume	9.3 cm3/dose		Assumed, based on RTS,S malaria vaccine
Doses in schedule	Single		Assumed, based on WHO Target Product Profile
Cost of commodities			
Cost of vaccine per dose, USD	$3 (range $1–5)		Assumption
Cost per administration syringe, USD	$0.06	$0.06	**Bangladesh**: EPI 2023**Nepal**: EPI and UNICEF purchase price 2023/2024
Cost per reconstitution syringe, USD	$0.06	$0.04	
Cost per safety box (100 syringe capacity), USD	$0.35	$0.50	
Procurement add-on charges as a percentage of product cost:			
Freight, insurance, inspection Handling fees Other taxes TOTAL	9% 4% 5% 18%	30% 5% 0% 35%	**Bangladesh**: EPI 2023**Nepal**: Calculate, based on EPI and UNICEF purchase price 2023/2024
Service delivery			
Percentage of vaccination in routine setting	20%	25%	**Bangladesh**: Assumed, informed by Ministry of Health (MOH) **Nepal**: Assumed, informed by MOH
Percentage of vaccination through outreach activities	80%	75%	
Average time to vaccinate one person (range), in-facility	3.23 minutes (2.5–7.5 minutes)	10.40 minutes (6.0–12.5 minutes)	Primary data collected during the health facility surveys
Average time to vaccinate one person (range), outreach	3.85 minutes (2.5–6.5 minutes)	13.7 minutes (8.5–17.5 minutes)	
Salaries and per diem rates			
Staff salary per month, range by staff cadre (in local currency units)	20,383 to 87,000 BGD	66,538 to 34,052 NRS	Bangladesh: Government Circular, 15 Dec 2015, effective from 1 July 2015. Provided by EPI. Nepal: Public service commission salary scale 2079 BS (2022).
Vaccinators’ average salary per month	21,117 BGD	40,581 NRS	Assumes: Grade 16 Family welfare Assistant (Bangladesh); Grade level 4 Nurses (Nepal)
Per diem rate per day, range	600 – 2,500 BGD	1,200 – 1,600 NRS	Bangladesh: Guideline from MOHFW (2017) Nepal: Guideline from MOH (2023)
Others			
Useful life years for capital investments	10		Assumed [22]
Useful life years of introduction activities	5		
Discount rate	3%		
Exchange rate [1 USD =]	109 BDT	131.86 NRS	**Bangladesh:** Oanda, October 2023 **Nepal:** Oanda, December 2023 [23]

Data collection occurred between August and November 2023 in Bangladesh and June and December 2023 in Nepal. Cost data were collected in local currency units (LCU) and presented in LCU and United States dollar (USD) 2023 units.

The study was approved by the Bangladesh Medical Research Council (BMRC) ethics review committee in Bangladesh, and the Nepal Health Research Council (NHRC) and BP Koirala Institute of Health Sciences (BPKIHS) ethics review committees in Nepal. All study participants provided written informed consent to participate in the study prior to their participation.

### Capacity considerations and allocation of shared resources to MI

Since new maternal vaccines are not yet widely used in LMICs, characteristics of the future product and the potential cold chain capacity requirements are unknown. The currently licensed RSV maternal vaccine [23] is a single dose presentation. A multidose vial option is in development for the Gavi LMIC market. In the absence of an available product for the LMIC context, we assumed a three-doses per vial (Table 1) and the anticipated dose quantity to estimate the cold chain storage requirements for costing purposes.

Both countries have established vaccine distribution systems. To account for the incremental recurring costs of distribution, we estimated the total cost of vaccine delivery currently incurred annually based on data derived from administrative and financial records. Incremental contributions to future MI delivery were then valued at 10% (for baseline cost estimates) of the total distribution cost based on direct allocation. The same allocation factor was attributed to MI for all other joint operations activities such as routine supervision and monitoring. We estimated the incremental time required to administer a maternal vaccine dose based on average vaccination time for TTCV as reported by health workers during the health facility survey (*N* = 20 Bangladesh and *N* = 5 Nepal).

### Study outcomes

Annual cost projections for each activity are calculated for the study period (five years) to generate total costs of the program. These costs are divided into introduction and recurrent costs. Introduction costs are divided by the total target population over the study period to generate introduction related unit costs. Recurrent costs over the study period are divided by the expected number of doses delivered to estimate the recurring unit cost per dose. The sum of introduction and recurrent costs is the cost per dose administered. MI delivery costs are calculated with and without considering the costs associated with vaccine and immunization supplies.

## Results

### Expected number of vaccinations and total costs

All pregnant women over a five-year study period (21.2 million in Bangladesh and 2.6 million in Nepal) were assumed eligible to receive a single dose of maternal vaccine. Using baseline vaccine coverage (Table 1), 19.1 million and 1.9 million pregnant vaccine recipients were projected to be vaccinated over 5 years in Bangladesh and Nepal, respectively.

At a baseline vaccine price of $3 per dose, the total estimated cost (financial) of a MI program is $5.2 million in Bangladesh and $1.4 million in Nepal, annually. Similarly, the total estimated annual economic cost of MI is $24.6 million and $3.8 million in Bangladesh and Nepal, respectively. Since the vaccine is assumed to be donated, it is included only in the economic cost estimates. The cost of other immunization supplies and the procurement add-on cost of vaccine and immunization supplies are assumed to be paid by the government and are included in financial costs. Attribution of these costs to initial set-up (introduction) and recurrent operational activities are presented in Table 2.

**Table 2:** Target population, projected vaccinations, and projected total costs (USD) over a 5-year study period

Metric	Bangladesh		Nepal	
	All years	Annual average	All years	Annual average
Target population				
Total target population (pregnant women)	21,272,020	4,254,404	2,649,687	529,937
Total number of doses delivered	19,144,818	3,828,964	1,918,581	383,716
Total cost (in USD)				
Financial	26,077,397	5,215,479	7,292,890	1,458,578
Economic	123,094,812	24,618,962	19,412,845	3,882,569
Introduction cost, Financial	8,767,115	1,753,423	3,904,187	780,837
Introduction cost, Economic	14,001,807	2,800,361	6,859,143	1,371,829
Recurrent cost, Financial	17,310,282	3,462,056	3,388,703	677,741
Recurrent cost, Economic	109,093,005	21,818,601	12,553,702	2,510,740

### Unit cost projections

The projected unit cost estimates of MI delivery are presented in Table 3. The cost per eligible pregnant vaccine recipient in Bangladesh is estimated at $1.09 (financial) and $5.62 (economic) and in Nepal, $2.72 (financial) and $7.55 (economic). The financial introduction costs per target population are projected at $0.28 (Bangladesh) and $1.38 (Nepal). Similarly, the financial recurrent costs per dose administered are $0.90 (Bangladesh) and $1.85 (Nepal). Excluding costs associated with vaccine and related immunization commodities, the cost of MI delivery per dose administered is estimated at $0.45 and $1.81 (financial), and $2.01 and $4.49 (economic), in Bangladesh and Nepal, respectively.

**Table 3:** Unit cost estimates (in USD) of maternal immunization delivery in Bangladesh and Nepal

Metric	Bangladesh		Nepal	
	Financial	Economic	Financial	Economic
Cost per eligible target population^1^	1.09	5.62	2.66	7.47
Introduction cost per eligible target population^2^	0.28	0.49	1.38	2.73
Recurrent cost per dose administered^3^	0.90	5.69	1.77	6.54
Cost of delivery per dose, with commodities^4^	1.18	6.19	3.14	9.27
Cost of delivery per dose, excluding commodities^5^	0.45	2.01	1.81	4.49

^1^ *Total cost over 5 years divided by total target population (expected number of pregnant women)*

^2^ *Total introduction cost (initial set-up cost) divided it by total target population*

^3^ *Total recurring costs divided by the total number of doses administered*

^4^ *Total costs including immunization commodities: vaccine/injection supplies cost divided by total number of doses administered as relevant for financial and economic cost estimates*

^5^ *Total cost excluding cost related to immunization commodities: vaccine/injection supplies cost divided by total number of doses administered*

### Cost drivers

In Bangladesh, introduction costs make up a quarter of the total financial cost. Under baseline input assumptions, commodity procurement (vaccine and other immunization supplies) constitutes 60% and 67% of the total financial and economic costs, respectively. Note that the cost of vaccine is excluded from financial cost, but it includes the procurement add-on cost calculated as a percentage of vaccine cost. Excluding commodities, the major cost drivers of total financial cost are program planning and coordination (29%), demand creation (23%), health worker training (19%), and monitoring and evaluation activities (18%). For economic cost, program planning and coordination constitute the major cost driver (68%), excluding cost of commodities, followed by monitoring and evaluation (10%) and health workers training (8%).

Similarly, in Nepal, introduction costs constitute about half of the total financial costs. Procurement of immunization commodity accounts for 36% of the total financial cost and 46% of the total economic cost. Excluding commodities, the main cost driver of total cost is health worker training accounting for about 44% of both financial and economic cost. Program planning and coordination (15%) and demand creation activities (14%) were other major drivers of financial total costs. Other economic cost drivers include health worker training (44%), vaccine distribution (17%), program planning and coordination (12%), and demand creation and service delivery (9% each). Drivers of total cost (including cost of commodity) are provided in Tables 4 and 5.

**Table 4:**
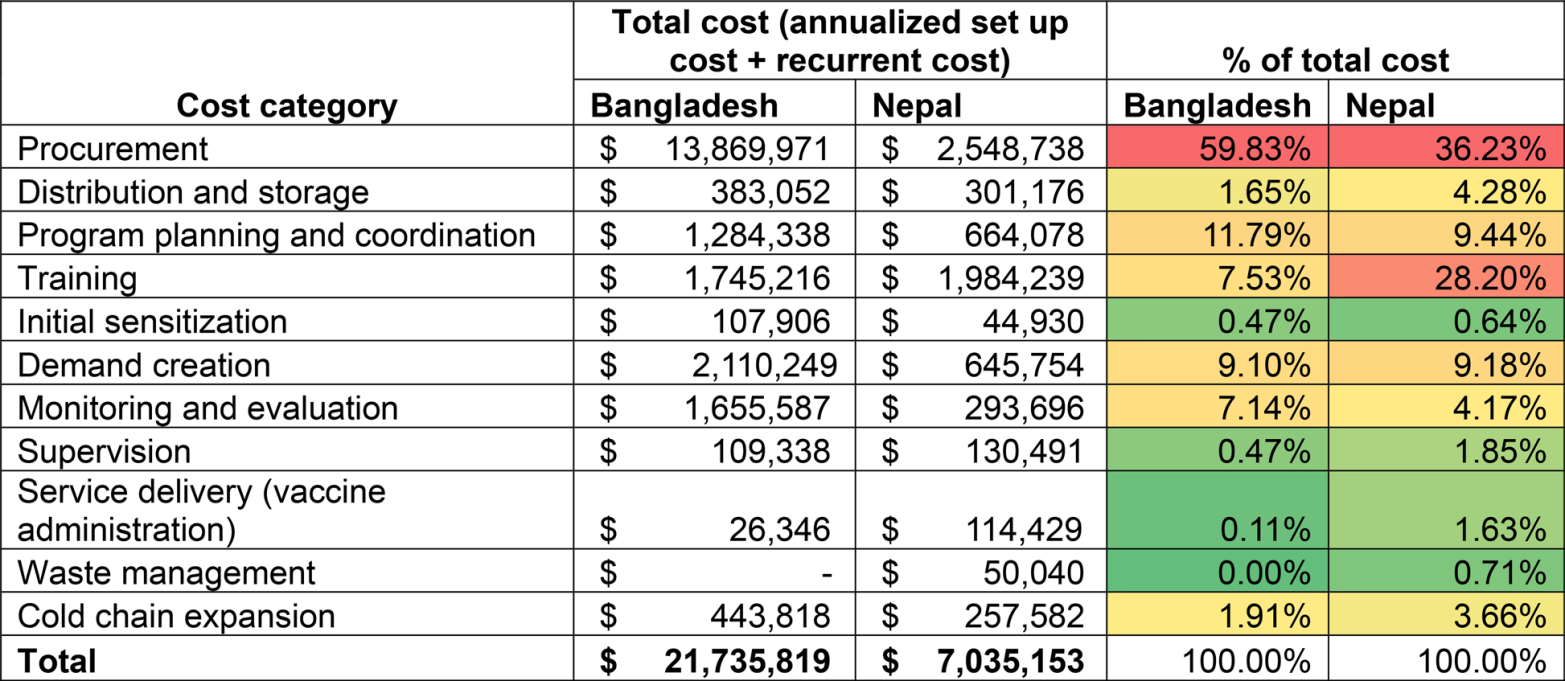
Financial cost of MI introduction and delivery in Bangladesh and Nepal (annualized set up cost and recurrent cost), 2024–2028 (in USD)

Financial cost of procurement include costs of injection supplies and shipping and handling associated cost of vaccine and immunization supplies. Red color code suggests major cost drivers and green color suggests non-major cost drivers of total cost. The cost share percentages are calculated as a percentage of total cost.

**Table 5:**
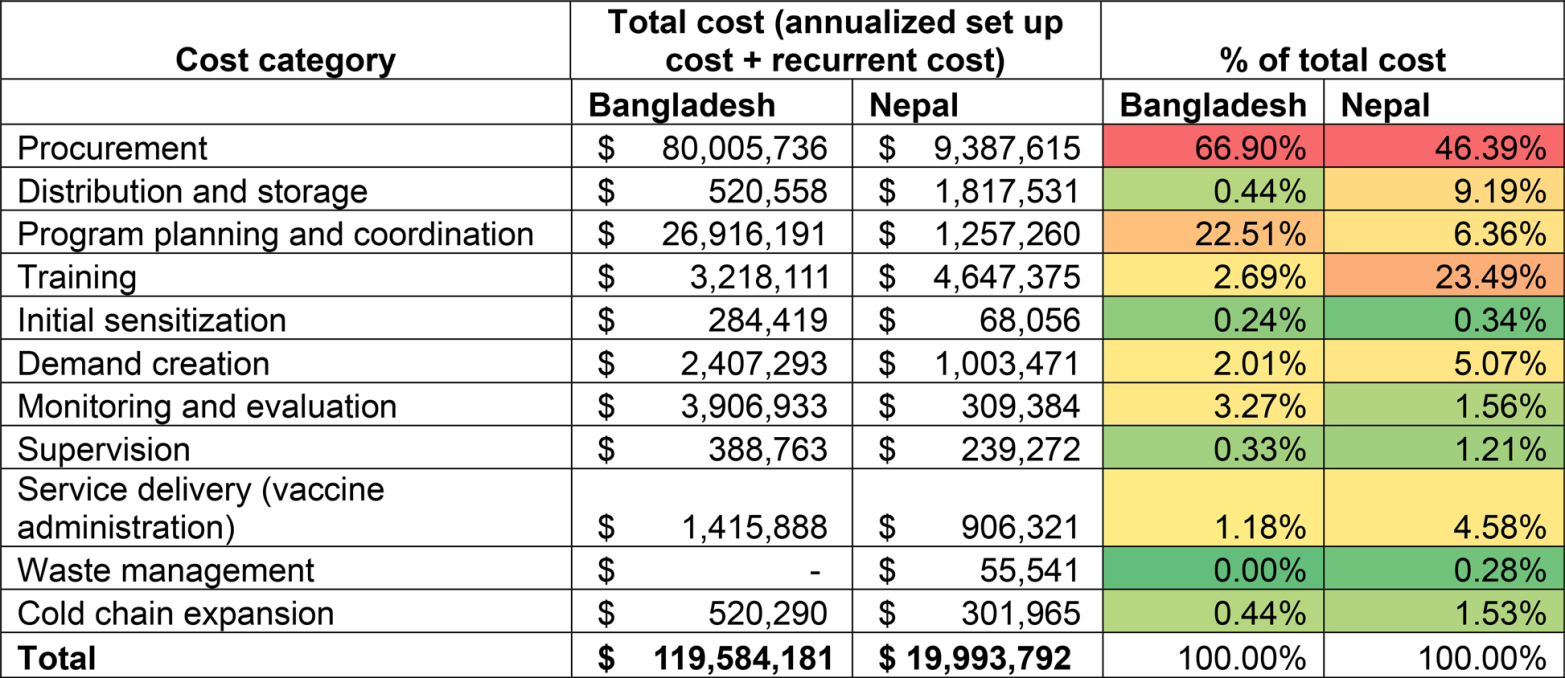
Economic cost of MI introduction and delivery in Bangladesh and Nepal (annualized set up cost and recurrent cost), 2024–2028 (in USD)

Economics cost of procurement include financial procurement costs plus cost of vaccine product (assumed to be donated to the government). Red color code suggests major cost drivers and green color suggests non-major cost drivers of total cost. The cost share percentages are calculated as a percentage of total cost.

Since the maternal vaccines considered in this analysis are not yet available for LMIC markets, we made several assumptions on future product inputs such as product prices, anticipated coverage rates, and other considerations. To evaluate the implication of these assumptions on the unit cost estimates, we did a one-way sensitivity analysis, and results are presented in the tornado diagrams in Appendix 3. The maternal vaccine product price assumption most greatly influences the financial and economic cost estimates of cost per dose administration. Under the vaccine price assumption of $1 and $5 per dose, the financial cost per dose (which accounts only for procurement add-on costs) ranges between $0.76 and $1.59 in Bangladesh and $2.34 and $3.95 in Nepal. Under the same assumption, the economic cost per dose ranges between $3.47 and $8.91 in Bangladesh and $6.16 and $12.38 in Nepal. Assumptions on vaccine coverage and allocation factor for shared resources are the other major inputs influencing the unit cost estimates (Appendix 3).

## Discussion

New maternal vaccines addressing major infectious disease burdens among pregnant populations and young children are on the horizon for LMICs. As new maternal vaccines become available, health systems may need adaptations and resources to create effective delivery mechanisms. In this paper, we project the costs of introducing and delivering new maternal vaccines in two south Asian country settings. Along with similar cost projections in several African country settings [15, 16] these cost projections help fill a gap in MI cost estimates for LMICs [14]. The cost estimates generated in this study can support new vaccine introduction decisions by providing country-specific cost projections reflecting the current health system capacity and resource needs. The utility of the study results also extends beyond country decision support by providing regional and global policymakers information on MI program costs and contexts that may inform future programs.

In this study, we use information on how pregnant populations receive vaccines to generate robust MI introduction and delivery cost estimates. In Bangladesh and Nepal, EPI programs are the major providers of TTCV to pregnant women. Pregnant women are typically referred to established EPI sessions for vaccination, which are held on fixed days each month to cover all immunization (childhood and pregnant women) in well-known locations within the health facility catchment area. Maternal, newborn, and child health (MNCH) service providers refer pregnant women to those sites for immunization. Improved coordination between the two programs is one of the major activities identified as needed in this study to support a conducive MI delivery platform. In both countries, improved planning and coordination in the initial phases of program introduction, and training of all health workers engaged in vaccination (including those from MNCH programs providing ANC services) are also identified as critical for effective MI delivery.

We generated financial and economic cost estimates from the health system perspective. Economic costs capture direct financial costs and other opportunity costs of resources like existing staff salaries and donated vaccines. The economic costs presented, therefore, include health workers’ time expected for MI introduction and delivery activities such as program planning and coordination, training, communication and social mobilization, and vaccine administration. For vaccine storage and distribution in both countries, no substantial infrastructure adaptation was identified as required outside of strengthening the cold chain capacity to accommodate new maternal vaccine storage.

Cost projections are influenced by various contextual factors specific to the country, so making direct comparisons can be misleading. Our findings suggest that the financial costs of MI per eligible target pregnant population are $1.09 in Bangladesh and $2.72 in Nepal. The number of pregnant women eligible for vaccination are about five times higher in Bangladesh than in Nepal. The higher target population in Bangladesh lends some cost efficiencies, particularly from introduction and initial set-up costs, which mostly occur at the national level and are divided by a larger denominator. The unit costs are also influenced by the expected coverage rates with higher coverage distributing the costs over larger population denominators, resulting in smaller unit cost estimates. This was partly reflected in the lower unit cost estimates in Bangladesh compared to Nepal. Many other factors such as salary scale and per diem rates also impact the unit cost projections. Both countries have relatively high anticipated coverage, averaging 90% in Bangladesh and 75% in Nepal. The ability of future MI programs to achieve higher coverage will ensure that the unit costs of such interventions remain relatively low.

The service delivery (vaccine administration) cost estimates in both countries primarily reflects the value of health workers’ time spent on vaccine delivery which was estimated using self-reported time data from the health facility survey. Limitations of self-reported time in valuing service delivery costs is acknowledged. Future estimates can be improved by using a time-motion study which was not considered in this study due to time- and resource-limitations. Also, in Bangladesh, multiple challenges with waste management (in particular high cost of installation and maintenance of incinerators in Bangladeshi low-land and the negative environmental consequences associated with alternative practices) were under active discussion with stakeholders during the period of data collection. Owing to the prevailing uncertainties, the costs associated with waste management were excluded from the analysis for Bangladesh. Although waste management typically does not constitute a major cost driver, this exclusion is a limitation of the present study.

Nepal, a landlocked country, has a much higher procurement add-on cost (e.g., shipping, handling of vaccine and immunization supplies) at 35% of the product cost, almost double that observed in Bangladesh (18%). The higher procurement add‑on cost led to relatively high immunization commodity procurement costs and higher estimates of financial and economic unit costs [Table 3]. Easing some of those costs via donor agency support would help alleviate the financial burden and affordability to LMICs. Currently, donor agencies partly support such costs in many countries.

This is the first study to project the cost of MI delivery in Bangladesh or Nepal and begins to address the current paucity of immunization COD estimates in LMICs. One recent study on COVID-19 vaccine delivery to the general population in Bangladesh [24] reports the non‑vaccine financial cost of delivery to be between $0.29 at MOH hospitals and $0.44 at outreach EPI centers. The economic costs of delivering a dose of COVID-19 vaccine are reported to range between $0.74 and $2.15. Our MI COD estimates (excluding commodity cost) for Bangladesh are similar to delivery costs for COVID-19 vaccine [24]. The convergence of these cost projections could be explained by the fact that the MI delivery mechanism is anticipated to largely leverage existing vaccine delivery mechanisms in countries. A separate study modeling the cost of delivering childhood vaccination projected the economic cost of delivery at $2.05 (95% uncertainty interval [UI] $0.65 and $5.04) in Bangladesh and $2.00 (95% UI $0.79 and $4.30) in Nepal [25]. Our cost projections are within the modeled cost projection range for both countries [25], suggesting alignment between MI and other new childhood vaccine delivery costs.

The cost projection generated in this study builds on anticipated activities and resource needs for future MI introduction, based on discussions with country decision-makers. Comparing these cost projections with costs observed during actual implementation of MI in the future in a few geographies will enable validation of the costs presented herein. Variations between the two estimates can offer insights into the need for undertaking similar cost projections in other settings. These insights are important as country decision-makers setting public health priorities and intervention choices are faced with affordability questions for the sustainability of such interventions.

Since new MI products for LMICs are not yet widely available, this prospective costing study used assumptions informed by country program experts, enabling robust and realistic cost projections for each country. The cost projections, therefore, reflect current country capacity, gaps in capacity, and resources needed to support an effective country MI program. As such, we believe our projections provide useful country-specific insights to help inform affordability and economic feasibility of future MI interventions in these countries.

## Supplementary Information

Supplementary Material 1.

Supplementary Material 2.

## Data Availability

All data generated or analysed during this study are included in this published article [and its supplementary information files].
