# Supplementary material for: Maternal vaccine delivery costs in South Asian settings: estimates from Bangladesh and Nepal
Source: BMC Public Health. 2025 Dec 3;26:98. doi: 10.1186/s12889-025-25786-3 (PMC12781477; doi:10.1186/s12889-025-25786-3)
Supplement: Supplementary file 1 — Supplementary Material 1. [file 12889_2025_25786_MOESM1_ESM.docx]

**Supplementary materials**

#### **Appendix Table 1: Detailed activities included in the costing analysis**

**Bangladesh**

| **Activities** | **Frequency of activities** |
| --- | --- |
| **Vaccine and injection supplies procurement** |  |
| Procurement of maternal vaccine doses | Each year |
| Procurement of supplies- reconstitution syringe | Each year |
| Procurement of supplies- administration syringe | Each year |
| Procurement of supplies- safety boxes | Each year |
| **Distribution** |  |
| Receive quarterly vaccine and immunization supplies at the national store | Quarterly (4 times) a year, shared cost |
| Distribution of vaccine and supplies from National store to district stores | Quarterly (4 times) a year, shared cost |
| Collection of vaccine and supplies from district vaccine store by Upazila stores | Monthly, shared cost |
| Distribution of vaccine and supplies from Upazila to union level/ward level | Monthly, shared cost |
| Collection of vaccine and supplies from district vaccine store by city corporation/zones | Monthly, shared cost |
| Collection of vaccine and supplies from by ward from zonal level | Monthly, shared cost |
| Dry storage space rental fees at national level | Monthly, shared cost |
| Operating cost of cold chain and maintenance at national, district, and Upazila levels | Monthly, shared cost |
| **Program planning and coordination** |  |
| Hold National Immunization Technical Advisory Group (NITAG) meeting to review policies and guidelines | Once per quarter in the introduction year only |
| Inter-Agency Coordination Committee meeting | Two meetings in the introduction year only |
| Vaccine Implementation Technical Coordination Committee meeting to plan and coordinate implementation | Monthly meeting in the introduction year only |
| Technical Sub-committee meetings: Planning, Coordination and Management sub-committee | Monthly meeting in the introduction year only |
| High level Inter-ministerial consultation meeting | Once in the introduction year |
| Coordination Committee meeting at division, city corporation, district and Upazila levels | Monthly meeting in the introduction year only |
| Microplanning Activity at national, Upazila, and health Facility levels | Once in the introduction year |
| Routine microplanning activity at national, Upazila, and health facility levels | Each year, shared cost |
| **Training** |  |
| Technical sub-committee meetings: training and surveillance | Five meetings in the introduction year only |
| Workshop to develop training package and tools | Once in the introduction year |
| Printing of training tools | Once in the introduction year |
| National level training of trainers (TOT) | Once in the introduction year |
| TOT for district participants | Once in the introduction year |
| TOT for city corporation participants | Once in the introduction year |
| District level training for Upazila team | Once in the introduction year |
| Upazila level training for health facility team (vaccinators) | Once in the introduction year |
| City corporation level training for Urban primary health care team (vaccinators) | Once in the introduction year |
| Health facility level training for ward-based team and volunteers | Once in the introduction year |
| Adverse event following immunization (AEFI) training for doctors and nurses in all district, city corporation, and Upazila levels | Once in the introduction year |
| AEFI management cost and AEFI tool kit for health facility | Once in the introduction year |
| **Initial sensitization** |  |
| National level advocacy meeting with all stakeholders | Once in the introduction year |
| Professional group sensitization | Once in the introduction year |
| Journalist and media sensitization / national press briefing | Once in the introduction year |
| Stakeholders’ sensitization at division, city corporation, municipality, district, and Upazila levels | Once in the introduction year |
| **Communication / demand creation** |  |
| Technical sub-committee meeting: Social mobilization and communication (including radio message, social media message, TV message, etc.) | Monthly meetings in the introduction year only |
| Information, Education, and Communication (IEC) material development workshop | Once in the introduction year |
| Printing of IEC materials and job aides for health workers | Once in the introduction year |
| Airing / broadcasting of communication materials (TV spots, radio spots, etc.) | Once in the introduction year |
| National launch event | Once in the introduction year |
| Launch events at districts and city corporation | Once in the introduction year |
| Press conference at national, district, and city corporation levels | Once in the introduction year |
| Orientation to community teachers, volunteers, and religious groups in Upazila and city corporation levels | Once in the introduction year |
| **Monitoring and evaluation** |  |
| Monitoring and evaluation sub-committee meeting | Monthly meetings in the introduction year only |
| Workshop for review and adaptation of monitoring and documentation tools (including AEFI) | Once in the introduction year |
| Printing and distribution of monitoring and evaluation tools | Once in the introduction year |
| Post introduction evaluation | Once in the introduction year |
| Monthly review meeting at district, city corporation, and Upazila levels | Each year, shared cost |
| **Supervision** |  |
| National level supervision pre-introduction for readiness | Once in the introduction year |
| National level supervision during launch | Once in the introduction year |
| City corporation level supervision | Once in the introduction year |
| Division and district level supervision | Once in the introduction year |
| Health facility level supervision | Once in the introduction year |
| **Service delivery** |  |
| Vaccination administration through routine Expanded Programme on Immunization or antenatal care clinic (fixed strategy) | Each year, shared cost |
| Vaccination administration through routine outreach | Each year, shared cost |
| **Cold chain procurement** |  |
| Cold room walk in (capacity: 40 cu m) added at national level | In introduction year only |
| Refrigerator (capacity: 240 l) added to all district vaccine stores | In introduction year only |
| Two cold box (capacity: 18 l) and 4 vaccine carriers (capacity: 3 l) added to each Upazila | In introduction year only |
| One cold box (capacity: 18 l) and 1 vaccine carrier (capacity: 3 l) added to each city corporation | In introduction year only |
| **Other capital equipment purchase** |  |
| None |  |

**Nepal**

| **Activities** | **Frequency of activities** |
| --- | --- |
| **Vaccine and injection supplies procurement** |  |
| Procurement of maternal vaccine doses | Each year |
| Procurement of supplies- reconstitution syringe | Each year |
| Procurement of supplies- administration syringe | Each year |
| Procurement of supplies- safety boxes | Each year |
| **Distribution** |  |
| Monthly receipt of vaccine and immunization supplies to the national store | Monthly, shared cost |
| Monthly distribution of vaccines from national to the provincial stores | Monthly (2 trips per month to cover all province), shared cost |
| Monthly distribution of vaccines from province to district vaccine stores | Monthly, shared cost |
| Monthly vaccine collection trips made by health facilities to district/cold chain points | Monthly, shared cost |
| Operating cost of cold chain and maintenance at all levels | Monthly, shared cost |
| **Program planning and coordination** |  |
| Hold National Immunization Advisory Committee Meeting | Four meetings in the introduction year only |
| Technical Working Committee Meetings: Planning and Coordination Committee | Six meetings in the introduction year only |
| Workshop to finalize introduction plan | One workshop in the introduction year only |
| Micro plan development/update at the national Level | Once in the introduction year |
| Micro plan development/update at the province Level | Once in the introduction year |
| Micro plan development/update at the district Level | Once in the introduction year |
| Annual microplanning at all levels | Each year, shared cost |
| **Training** |  |
| Technical sub-committee meeting: training and surveillance | Six meetings in the introduction year only |
| Workshop to develop training package and tools | Once in the introduction year |
| Printing of training tools | Once in the introduction year |
| National level training of trainers (TOT) for province officers | Once in the introduction year |
| TOT for district participants at province level | Once in the introduction year |
| District level training for health facilities | Once in the introduction year |
| Health facility level training for health workers | Once in the introduction year |
| **Initial sensitization** |  |
| [National level coordination meeting with various ministries](file:///C:\Users\rbaral\Box\MI%20Support\MI%20Support-INT\MI%20Support-INT-PAI\Activity%204\COD%20MODELS\Tables%20for%20manuscript_NEPAL.xlsx#RANGE!C4) | Once in the introduction year |
| [Sensitization of professional groups and private facility chiefs at national level](file:///C:\Users\rbaral\Box\MI%20Support\MI%20Support-INT\MI%20Support-INT-PAI\Activity%204\COD%20MODELS\Tables%20for%20manuscript_NEPAL.xlsx#RANGE!C39) | Once in the introduction year |
| [Sensitization of political leaders at national level](file:///C:\Users\rbaral\Box\MI%20Support\MI%20Support-INT\MI%20Support-INT-PAI\Activity%204\COD%20MODELS\Tables%20for%20manuscript_NEPAL.xlsx#RANGE!C74) | Once in the introduction year |
| Sensitization of political and religious leaders at province level | Once in the introduction year |
| Sensitization of political and religious leaders at district level | Once in the introduction year |
| Media sensitization at national level | Once in the introduction year |
| Media sensitization at province level | Once in the introduction year |
| Media sensitization at district level | Once in the introduction year |
| **Communication / demand creation** |  |
| Technical sub-committee meeting: social mobilization and communication (including radio message, social media message, TV message, etc.) | Six meetings in the introduction year only |
| Workshop to develop communication materials | Once in the introduction year |
| Printing of communication materials | Once in the introduction year |
| Production of messages for TV/radio/social media | Once in the introduction year |
| Airing of messages on TV, radio, and social media | Once in the introduction year |
| Mobilize communication van with maternal immunization messages | Once in the introduction year |
| Female community health volunteer orientation and sensitization at health facility level | Once in the introduction year |
| Community education (village leaders, mothers) on maternal immunization | Once in the introduction year |
| Launch introduction at national level | Once in the introduction year |
| Launch introduction at province level | Once in the introduction year |
| Launch introduction at district level | Once in the introduction year |
| **Monitoring and evaluation** |  |
| [Monitoring and evaluation sub-committee meeting](file:///C:\Users\rbaral\Box\MI%20Support\MI%20Support-INT\MI%20Support-INT-PAI\Activity%204\COD%20MODELS\Tables%20for%20manuscript_NEPAL.xlsx#RANGE!C4) | Six meetings in the introduction year only |
| [Modification of monitoring/reporting/recording tools](file:///C:\Users\rbaral\Box\MI%20Support\MI%20Support-INT\MI%20Support-INT-PAI\Activity%204\COD%20MODELS\Tables%20for%20manuscript_NEPAL.xlsx#RANGE!C39) | Once in the introduction year |
| [Post introduction evaluation](file:///C:\Users\rbaral\Box\MI%20Support\MI%20Support-INT\MI%20Support-INT-PAI\Activity%204\COD%20MODELS\Tables%20for%20manuscript_NEPAL.xlsx#RANGE!C109) | Once in the introduction year |
| [Printing of recording and monitoring tools](file:///C:\Users\rbaral\Box\MI%20Support\MI%20Support-INT\MI%20Support-INT-PAI\Activity%204\COD%20MODELS\Tables%20for%20manuscript_NEPAL.xlsx#RANGE!C144) | Each year, shared cost |
| **Supervision** |  |
| Pre-introduction supervision from national to province level | Once in the introduction year |
| Pre-introduction supervision from province to district level | Once in the introduction year |
| Pre-introduction supervision from district to health facility level | Once in the introduction year |
| Introduction supervision from national to province level | Once in the introduction year |
| Introduction supervision from province to district level | Once in the introduction year |
| Introduction supervision from district to health facility level | Once in the introduction year |
| Routine quarterly integrated supervision from national to province level | Four times each year, shared cost |
| Routine quarterly integrated supervision from province to district level | Four times each year, shared cost |
| Routine quarterly integrated supervision from district to health facility level | Four times each year, shared cost |
| **Service delivery** |  |
| Vaccination administration through routine Expanded Programme on Immunization (fixed site) | Each year, shared cost |
| Vaccination administration through routine outreach | Each year, shared cost |
| **Cold chain procurement** |  |
| Cold room walk in (capacity: 40 cu m) added at national level | In introduction year only |
| Refrigerator (capacity: 240 l) added to all district vaccine stores | In introduction year only |
| Refrigerator (capacity: 240 l) added to all province vaccine stores | In introduction year only |
| Five cold box (capacity: 18 l) and 5 vaccine carrier (capacity: 3 l) added per district to support health facilities | In introduction year only |
| **Waste management** |  |
| Procurement of plastic twin bucket | One per health facility, in introduction year only, shared cost |
| Construction of safety pits | Construct five pits per district, in introduction year only, shared cost |
| Purchase of waste collection bags (red and black bags) | Five per health facility each month, shared cost |
| Procure hub cutter for safe waste disposal | Two per health facility, in introduction year only, shared cost |

#### **Appendix Table 2: List of areas and facilities surveyed for costing**

**Bangladesh**

**Key informants’ interview**

1. National level: Chiefs and representatives from
   - Maternal Neonatal, Child and Adolescent Health program, Directorate General of Health Services (DGHS), Directorate General Family Planning (DGFP), National Immunization Program (EPI)
   - Program focal points
2. City corporation / districts: Civil surgeons, chief health officers, program representatives
3. Upazila: Health officers and representative focal points
4. Facilities surveys
   - 2 health facilities in each Upazila (N=16)
   - 1 health facility in each city corporation (N = 4)
5. Vaccine stores (N=15):
   - National level (N=1), District vaccine stores (N=4), Upazila vaccine stores (N=6), City corporation vaccine store (N=4)

Figure 2.1: Areas and facilities surveyed for costing in Bangladesh

**Nepal**

**Key informants’ interview**

1. National level: Chiefs and representatives from

- MNCH, EPI
- Program focal points

1. Provinces: Province health officers, program representatives
2. Districts: District health officers and representative focal points
3. Facility survey
   - Health facilities (N=5)
   - Vaccine stores (National level (N=1), Province vaccine stores (N=2), District vaccine stores (N=3))

Figure 2.2: Areas and facilities surveyed for costing in Nepal

#### **Appendix 3: One way sensitivity analysis**

**Figure 1: One way sensitivity of unit cost estimates for maternal immunization introduction and delivery in Bangladesh**

**Figure 2: One way sensitivity of unit cost estimates for maternal immunization introduction and delivery in Nepal**
