# Supplementary material for: Maternal vaccine delivery costs in South Asian settings: estimates from Bangladesh and Nepal
Source: BMC Public Health. 2025 Dec 3;26:98. doi: 10.1186/s12889-025-25786-3 (PMC12781477; doi:10.1186/s12889-025-25786-3)
Supplement: Supplementary file 2 — Supplementary Material 2. [file 12889_2025_25786_MOESM2_ESM.docx]

**Maternal Immunization (MI) INTRODUCTION AND DeliverY COST**

| **O. General information** | |
| --- | --- |
| **County name:** | **Province/district name:** |
| **Facility name:** | **Facility type/level:** |
| **Name/designation/department/contact** | **Date:** |
| **Interviewee 1:** |  |
| **Interviewee 2:** |  |
| **Interviewee 3:** |  |

| **I.1** | **General information** | **Responses** |
| --- | --- | --- |
| I.1.1 | What is the target population served by this health facility's catchment area? |  |
| I.1.2 | How many children are in the target population for the EPI program (under 1 year) in this health facility’s catchment area? |  |
| I.1.3 | How many pregnant women are in the target population for the MNCH program in this health facility’s catchment area? |  |
| I.1.4 | What are the various ways in which immunization services to children are delivered at this facility? Include by whom, and where, the vaccines are administered? |  |
| I.1.5 | What are the various ways in which immunization services to pregnant women are delivered at this facility? Include by whom, and where, the vaccines are administered? |  |
| **I.2** | **Fixed immunization sessions** |  |
| I.2.1 | How often are immunization services held at this facility, where the caregivers bring the children to this health facility to receive immunizations? |  |
| I.2.2 | On a typical immunization session day when you provide immunization services at this facility, what time do you start and end the sessions? |  |
| I.2.3 | On the days when immunization services are provided at this facility, are other services also provided? |  |
| I.2.4 | Is antenatal care (ANC) also provided together with fixed immunization services? |  |
| I.2.5 | How many people do you usually serve on one fixed immunization session? |  |
| I.2.6 | How many children do you usually vaccinate (for all antigens) on one fixed immunization session? |  |
| I.2.7 | How many pregnant women do you usually vaccinate in one fixed immunization session? |  |
| I.2.8 | How long does it take for you to vaccinate one child during a static/fixed session? *(Please include time spent including preparing for vaccines, vaccination, filling out tally sheet, etc.)* |  |
| I.2.9 | How long does it take for you to vaccinate one pregnant woman during a static/fixed session? (Please include time spent including preparing for vaccines, vaccination, filling out tally sheet, etc.) |  |
| **I.3** | **Outreach/mobile immunization sessions** |  |
| I.3.1 | Does this health facility conduct routine outreach or mobile immunization sessions? |  |
| I.3.2 | How often are outreach or mobile immunization sessions conducted by staff from this health facility? |  |

| **For each routine outreach session conducted by this facility, list the following** | | | | | | | | |
| --- | --- | --- | --- | --- | --- | --- | --- | --- |
| ***Outreach location*** | ***Outreach frequency per month*** | ***Distance from this facility*** | ***Means of transportation*** | ***Number of staff traveling per session*** | ***Number of volunteers per session*** | ***Round trip transportation cost per person*** | ***Allowance paid per staff*** | ***Allowance paid per volunteer*** |
|  |  |  |  |  |  |  |  |  |
|  |  |  |  |  |  |  |  |  |
|  |  |  |  |  |  |  |  |  |
|  |  |  |  |  |  |  |  |  |
|  |  |  |  |  |  |  |  |  |
|  |  |  |  |  |  |  |  |  |
|  |  |  |  |  |  |  |  |  |
| **add rows as needed** |  |  |  |  |  |  |  |  |

| I.3.3 | On the days when outreach or mobile immunization services are provided, are other services also provided? | | | | | | |  | | | |  |
| --- | --- | --- | --- | --- | --- | --- | --- | --- | --- | --- | --- | --- |
| I.3.4 | Is antenatal care (ANC) also provided together with outreach or mobile immunization services? | | | | | | |  | | | |  |
| I.3.5 | How many people do you usually serve on each outreach session? | | | | | | |  | | | |  |
| I.3.6 | How many children do you usually vaccinate (for all antigens) on one outreach immunization session? | | | | | | |  | | | |  |
| I.3.7 | How many pregnant women do you usually vaccinate in one outreach or mobile immunization session? | | | | | | |  | | | |  |
| I.3.8 | How long does it take for you to vaccinate one child in outreach session? (*Please include time spent including preparing for vaccines, vaccination, filling out tally sheet, etc.*) | | | | | | |  | | | |  |
| I.3.9 | How long does it take for you to vaccinate one pregnant woman during an outreach or mobile session? (*Please include time spent including preparing for vaccines, vaccination, filling out tally sheet, etc.*) | | | | | | |  | | | |  |
| **I.4** | **Campaign immunization sessions** | | | | | | |  | | | |  |
| I.4.1 | Has this health facility organized campaign for any antigen in the last 3 year? | | | | | | |  | | | |  |
| I.4.2 | How is a campaign typically organized? Describe. If yes to IV.7.1, ask the respondent to provide information based on those campaigns. If ‘no’, ask respondent to provide estimates based on their experience. *(Use a separate sheet to record descriptive response as necessary)* | | | | | | |  | | | |  |
| **For each campaign conducted by this facility in the last 3 years list the following** | | | | | | | | | | | | |
| ***Target antigen*** | | ***Date /duration*** | ***Campaign location*** | ***Distance from facility*** | ***Means of transportation*** | ***Number of staff traveling per session*** | ***Number of volunteers per session*** | | ***Allowance per staff per day*** | ***Allowance paid per volunteer per day*** | ***Round trip transportation cost per person*** | |
|  | |  |  |  |  |  |  | |  |  |  | |
|  | |  |  |  |  |  |  | |  |  |  | |
|  | |  |  |  |  |  |  | |  |  |  | |
| **add rows as needed** | |  |  |  |  |  |  | |  |  |  | |

| I.4.3 | | How long (time in minutes) does it take for you to vaccinate one child in campaign session? *(Please include time spent including preparing for vaccines, vaccination, filling out tally sheet, etc.)* | | | | | | | | | | | | | | | | | | | | | | | | | | | | | | | | | |  | | | | | | | | | | | |
| --- | --- | --- | --- | --- | --- | --- | --- | --- | --- | --- | --- | --- | --- | --- | --- | --- | --- | --- | --- | --- | --- | --- | --- | --- | --- | --- | --- | --- | --- | --- | --- | --- | --- | --- | --- | --- | --- | --- | --- | --- | --- | --- | --- | --- | --- | --- | --- |
| I.4.4 | | How long (time in minutes) does it take for you to vaccinate one pregnant woman in campaign session? *(Please include time spent including preparing for vaccines, vaccination, filling out tally sheet, etc.)* | | | | | | | | | | | | | | | | | | | | | | | | | | | | | | | | | |  | | | | | | | | | | | |
| **I.5** | | **Potential service delivery models for MI interventions** | | | | | | | | | | | | | | | | | | | | | | | | | | | | | | | | | |  | | | | | | | | | | | |
| I.5.1 | | If this facility is to deliver MI vaccines to pregnant women, how would you organize the immunization session?  *(As an example of future maternal vaccine, describe the potential RSV maternal vaccine, and their potential use case to the facility and ask how they think would be the most feasible way to deliver this vaccine. Allow EPI and MNCH representatives to discuss and deliberate. Use separate sheet of paper to record the response as necessary.)* | | | | | | | | | | | | | | | | | | | | | | | | | | | | | | | | | |  | | | | | | | | | | | |
| **I.6** | | **Current practices on vaccination of pregnant women** | | | | | | | | | | | | | | | | | | | | | | | | | | | | | | | | | |  | | | | | | | | | | | |
| I.6.1 | | Please check one that describes most accurately how the vaccines currently being administered to pregnant women (e.g., TT) are delivered in this facility? | | | | | | | | | | | | | | | | | | | | | | | | | | | | | | | | | |  | | | | | | | | | | | |
| *(i)* | | *Pregnant women are vaccinated by EPI staff at routine EPI clinics on same days/time as routine childhood vaccination.* | | | | | | | | | | | | | | | | | | | | | | | | | | | | | | | | | |  | | | | | | | | | | | |
| *(ii)* | | *Pregnant women are vaccinated by EPI staff at routine EPI clinics on separate days/time as routine childhood vaccination.* | | | | | | | | | | | | | | | | | | | | | | | | | | | | | | | | | |  | | | | | | | | | | | |
| *(iii)* | | *Pregnant women are vaccinated by ANC staff on separate location/time (ANC clinic).* | | | | | | | | | | | | | | | | | | | | | | | | | | | | | | | | | |  | | | | | | | | | | | |
| *(iv)* | | *If others, describe:* | | | | | | | | | | | | | | | | | | | | | | | | | | | | | | | | | |  | | | | | | | | | | | |
| I.6.2 | | If answer to I.6.1 is (iii) and/or (iv), please describe the various ways in which immunization services to pregnant women are delivered at this facility? Include by whom, and where, the vaccines are administered? | | | | | | | | | | | | | | | | | | | | | | | | | | | | | | | | | |  | | | | | | | | | | | |
| I.6.3 | | How do pregnant women who do not attend ANC at health facility get vaccinated? | | | | | | | | | | | | | | | | | | | | | | | | | | | | | | | | | |  | | | | | | | | | | | |
| **II.** | | **Cold chain and storage** | | | | | | | | | | | | | | | | | | | | | | | | | | | | | | | | | |  | | | | | | | | | | | |
| II.1 | | Current capacity and need assessment for additional cold chain requirement | | | | | | | | | | | | | | | | | | | | | | | | | | | | | | | | | | | | | | | | | | | | | |
| II.1.1 | | Where are the maternal vaccines currently used in this facility (e.g., TD) usually stored? | | | | | | | | | | | | | | | | | | | | | | | | | | | | | | | | | |  | | | | | | | | | | | |
| II.1.2 | | What is the current total vaccine/cold chain storage capacity at this facility? (Volume, in cm3) | | | | | | | | | | | | | | | | | | | | | | | | | | | | | | | | | |  | | | | | | | | | | | |
| II.1.3 | | What proportion of vaccine storage capacity is occupied on average at this facility? | | | | | | | | | | | | | | | | | | | | | | | | | | | | | | | | | |  | | | | | | | | | | | |
| II.1.4 | | Does vaccine storage capacity in this facility vary across season? | | | | | | | | | | | | | | | | | | | | | | | | | | | | | | | | | |  | | | | | | | | | | | |
| II.1.5 | | Does this facility have spare cold chain storage capacity? If yes, please provide an estimate of the spare capacity? | | | | | | | | | | | | | | | | | | | | | | | | | | | | | | | | | |  | | | | | | | | | | | |
| II.1.6 | | Does this facility have adequate system to accommodate additional vaccines for new vaccine introduction? | | | | | | | | | | | | | | | | | | | | | | | | | | | | | | | | | |  | | | | | | | | | | | |
| II.1.7 | | Does the ANC clinic at this health facility have the capacity to store and distribute vaccines? | | | | | | | | | | | | | | | | | | | | | | | | | | | | | | | | | |  | | | | | | | | | | | |
| II.1.8 | | What specific cold chain equipment and storage facilities would be needed at this facility to introduce MI interventions? | | | | | | | | | | | | | | | | | | | | | | | | | | | | | | | | | |  | | | | | | | | | | | |
| II.1.9 | | Where would these additional cold chain equipment/facilities be added? For example, in ANC clinic, EPI clinic, outreach…? | | | | | | | | | | | | | | | | | | | | | | | | | | | | | | | | | |  | | | | | | | | | | | |
| II.1.10 | | During vaccination campaigns, how are the cold chain requirements typically handled? Describe. *(Please consider how this was done during a recent vaccination campaign)* | | | | | | | | | | | | | | | | | | | | | | | | | | | | | | | | | |  | | | | | | | | | | | |
| II.2 | | Cost of cold chain operation and maintenance *For each of the questions below, ask if there are costs incurred to the EPI and MNCH units, separately.* | | | | | | | | | | | | | | | | | | | | | | | | | | | | | | | | | | | | | | | | | | | | | |
| II.2.1 | | What is the total fuel and energy cost of operating the cold chain for vaccine storage in this facility, in the last year [Specify year: ………………….]? | | | | | | | | | | | | | | | | | | | | | | | | | | | | | | | | | | EPI unit:  MNCH unit: | | | | | | | | | | | |
| II.2.2 | | What is the total fuel and energy cost of operating the cold chain for immunization supplies for storage in this facility/vaccine store, in the last year? | | | | | | | | | | | | | | | | | | | | | | | | | | | | | | | | | | EPI unit:  MNCH unit: | | | | | | | | | | | |
| II.2.3 | | Was there any routine maintenance of cold chain equipment at this facility in the last one year? If yes, how much was paid in total in the last year for the routine maintenance of the cold chain equipment? | | | | | | | | | | | | | | | | | | | | | | | | | | | | | | | | | | EPI unit:  MNCH unit: | | | | | | | | | | | |
| II.3 | | Roles and responsibilities among staff currently working on EPI and MNCH | | | | | | | | | | | | | | | | | | | | | | | | | | | | | | | | | | | | | | | | | | | | | |
| II.3.1 | | How would roles and responsibilities work or need to change across EPI and MNCH staff responsible for cold chain and storage management, if any, for the introduction and delivery of MI interventions? *Please note responses separately for different scenarios, as applicable.* | | | | | | | | | | | | | | | | | | | | | | | | | | | | | | | | | |  | | | | | | | | | | | |
| II.4 | | Cold chain storage system in the MNCH units for maternal vaccine delivery | | | | | | | | | | | | | | | | | | | | | | | | | | | | | | | | | | | | | | | | | | | | | |
| II.4.1 | | Would there be need to install or update cold chain storage system in the MNCH units for maternal vaccine delivery? If yes, please describe the needs. | | | | | | | | | | | | | | | | | | | | | | | | | | | | | | | | | |  | | | | | | | | | | | |
| II.5 | | What are the biggest opportunities and challenges you foresee around MI intervention cold chain management? | | | | | | | | | | | | | | | | | | | | | | | | | | | | | | | | | |  | | | | | | | | | | | |
| **III.** | | **Distribution (supply chain management)** | | | | | | | | | | | | | | | | | | | | | | | | | | | | | | | | | |  | | | | | | | | | | | |
| III.1 | | Current capacity of vaccine and immunization supplies distribution [EPI] | | | | | | | | | | | | | | | | | | | | | | | | | | | | | | | | | | | | | | | | | | | | | |
| III.1.1 | | How are vaccines typically supplied (or collected) to this facility? | | | | | | | | | | | | | | | | | | | | | | | | | | | | | | | | | |  | | | | | | | | | | | |
| III.1.2 | | How are immunization supplies (syringes, etc.) typically supplied (or collected) to this facility? | | | | | | | | | | | | | | | | | | | | | | | | | | | | | | | | | |  | | | | | | | | | | | |
| III.1.3 | | How frequently are vaccines and immunization supplies, typically supplied (or collected) to this facility? | | | | | | | | | | | | | | | | | | | | | | | | | | | | | | | | | |  | | | | | | | | | | | |
| III.1.4 | | How many staff from this facility travel on each trip to collect/distribute vaccines for this level? | | | | | | | | | | | | | | | | | | | | | | | | | | | | | | | | | |  | | | | | | | | | | | |
| III.1.5 | | Are per diems paid for trips to collect vaccines? If yes, what is the amount of per diem per person per trip? | | | | | | | | | | | | | | | | | | | | | | | | | | | | | | | | | |  | | | | | | | | | | | |
| III.1.6 | | What mode of transportation is used to collect vaccine and immunization supplies to this facility store? Describe. | | | | | | | | | | | | | | | | | | | | | | | | | | | | | | | | | |  | | | | | | | | | | | |
| III.1.7 | | What is the estimated cost of transportation per trip? Describe how you reach at that estimate. | | | | | | | | | | | | | | | | | | | | | | | | | | | | | | | | | |  | | | | | | | | | | | |
| III.1.8 | | Is a hired vehicle utilized to collect/distribute vaccine and immunization supplies? If yes to III.1.13, what is the round-trip cost per hired vehicle for each trip? Please collect the details. | | | | | | | | | | | | | | | | | | | | | | | | | | | | | | | | | |  | | | | | | | | | | | |
| **Round trip cost per hired vehicle** | | | | | | | | | | | | | | | | | | | | | | | | | | | | | | | | | | | | | | | | | | | | | | | |
| **Route** | | | | | | | | **Number of …......... (*next level name*) covered** | | | | | | **Average distance travelled one way** | | | | | | | | **Amount paid per trip** | | | | | | | **Number of trips per year** | | | | | | **Volume transported in this trip** | | | | | | | | | **Notes/comments** | | | |
|  | | | | | | | |  | | | | | |  | | | | | | | |  | | | | | | |  | | | | | |  | | | | | | | | |  | | | |
|  | | | | | | | |  | | | | | |  | | | | | | | |  | | | | | | |  | | | | | |  | | | | | | | | |  | | | |
|  | | | | | | | |  | | | | | |  | | | | | | | |  | | | | | | |  | | | | | |  | | | | | | | | |  | | | |
| **add rows as needed** | | | | | | | |  | | | | | |  | | | | | | | |  | | | | | | |  | | | | | |  | | | | | | | | |  | | | |
| III.1.9 | | Is government vehicle utilized for vaccine and immunization supplies distribution? If yes, please collect the details. | | | | | | | | | | | | | | | | | | | | | | | | | | | | | | | | | |  | | | | | | | | | | | |
| **Government vehicle used for transportation** | | | | | | | | | | | | | | | | | | | | | | | | | | | | | | | | | | | | | | | | | | | | | | | |
| **Vehicle type** *(please include volume for refrigerated vehicle (in meter3)* | | | **Route** | | | | | | | **# of trips per month in this route** | | | | | **Round trip distance per trip on this route** | | | **# of days per round trip** | | | | | | **# of staff who travel on each trip** | | | | | | **Per diem per person** | | | | | | | **Fuel cost per round trip on this route** | | | | | | | **Notes/comments** | | | |
|  | | |  | | | | | | |  | | | | |  | | |  | | | | | |  | | | | | |  | | | | | | |  | | | | | | |  | | | |
|  | | |  | | | | | | |  | | | | |  | | |  | | | | | |  | | | | | |  | | | | | | |  | | | | | | |  | | | |
| **add rows as needed** | | |  | | | | | | |  | | | | |  | | |  | | | | | |  | | | | | |  | | | | | | |  | | | | | | |  | | | |
| III.1. 10 | | Is any other means of transport (public….) utilized for vaccine and immunization supplies distribution/collection at this level? Describe. | | | | | | | | | | | | | | | | | | | | | | | | | | | | | | | | | |  | | | | | | | | | | |  |
| III.1. 11 | | If yes, to III.1.10, please collect the details. | | | | | | | | | | | | | | | | | | | | | | | | | | | | | | | | | |  | | | | | | | | | | |  |
| **Cost of using public transportation** | | | | | | | | | | | | | | | | | | | | | | | | | | | | | | | | | | | | | | | | | | | | | | |  |
| **Mode of transportation** | | | | **Round trip distance per trip (average) using this mode** | | | | | | | **# of days per round trip** | | | | | | **# of trips per month using this mode** | | | **# of staff who travel on each trip** | | | | | **Per diem per person** | | | | | | **Transportation cost/fare per person for a round trip** | | | | | | | | **Fuel cost per round trip** | | | | | | **Notes/comments** | |  |
|  | | | |  | | | | | | |  | | | | | |  | | |  | | | | |  | | | | | |  | | | | | | | |  | | | | | |  | |  |
|  | | | |  | | | | | | |  | | | | | |  | | |  | | | | |  | | | | | |  | | | | | | | |  | | | | | |  | |  |
|  | | | |  | | | | | | |  | | | | | |  | | |  | | | | |  | | | | | |  | | | | | | | |  | | | | | |  | |  |
| III.2 | | Current capacity of maternal and child health related commodities/supplies distribution at this level [MNCH] | | | | | | | | | | | | | | | | | | | | | | | | | | | | | | | | | | | | | | | | | | | |  |  |
| III.2.1 | | How are commodities for maternal and child health (including ANC) typically supplied (or collected) at this facility store? Specify the store/unit at this level where these commodities are stored and distributed from. Also, specify the types of commodities. | | | | | | | | | | | | | | | | | | | | | | | | | | | | | | | | | |  | | | | | | | | | |  |  |
| III.2.2 | | How frequently are the MNCH related commodities typically supplied to this facility? | | | | | | | | | | | | | | | | | | | | | | | | | | | | | | | | | |  | | | | | | | | | |  |  |
| III.2.3 | | How are the MNCH related commodities distributed from this facility to the next level? Specify the level. | | | | | | | | | | | | | | | | | | | | | | | | | | | | | | | | | |  | | | | | | | | | |  |  |
| III.2.4 | | Is a hired vehicle utilized for MNCH related commodities supplies distribution? If yes, what is the round-trip cost per hired vehicle for each trip? Please collect the details. | | | | | | | | | | | | | | | | | | | | | | | | | | | | | | | | | |  | | | | | | | | | |  |  |
| **Round trip cost per hired vehicle** | | | | | | | | | | | | | | | | | | | | | | | | | | | | | | | | | | | | | | | | | | | | |  |  |  |
| **Route** | | | | | | **Number of …......... (*next level name*) covered** | | | | | | **Average distance travelled one way** | | | | | | **Amount paid per trip** | | | | | | | | | **Number of trips per year** | | | | | | **Volume transported in this trip** | | | | | | | **Notes/comments** | | | | |  |  |  |
|  | | | | | |  | | | | | |  | | | | | |  | | | | | | | | |  | | | | | |  | | | | | | |  | | | | |  |  |  |
|  | | | | | |  | | | | | |  | | | | | |  | | | | | | | | |  | | | | | |  | | | | | | |  | | | | |  |  |  |
|  | | | | | |  | | | | | |  | | | | | |  | | | | | | | | |  | | | | | |  | | | | | | |  | | | | |  |  |  |
| III.2.5 | | Is government vehicle utilized for MNCH related commodities distribution? If yes, please collect the details. | | | | | | | | | | | | | | | | | | | | | | | | | | | | | | | | | |  | | | | | | | | | |  |  |
| **Government vehicle used for transportation** | | | | | | | | | | | | | | | | | | | | | | | | | | | | | | | | | | | | | | | | | | | | | |  |  |
| **Vehicle type** *(please include volume for refrigerated vehicle (in meter3)* | | | | | | **Route** | | | **# of trips per month in this route** | | | | | | **Round trip distance per trip on this route** | | | | | | | | **# of days per round trip** | | | | **# of staff who travel on each trip** | | | | | | **Per diem per person** | | | | | **Fuel cost per round trip on this route** | | | | **Notes/comments** | | | |  |  |
|  | | | | | |  | | |  | | | | | |  | | | | | | | |  | | | |  | | | | | |  | | | | |  | | | |  | | | |  |  |
|  | | | | | |  | | |  | | | | | |  | | | | | | | |  | | | |  | | | | | |  | | | | |  | | | |  | | | |  |  |
|  | | | | | |  | | |  | | | | | |  | | | | | | | |  | | | |  | | | | | |  | | | | |  | | | |  | | | |  |  |
| III.2. 6 | | Is any other means of transport (public….) utilized MNCH related commodity distribution/collection at this level? Describe. Please collect the details. | | | | | | | | | | | | | | | | | | | | | | | | | | | | | | | | | |  | | | | | | | | | |  |  |
| **Cost of using public transportation per trip (round trip)** | | | | | | | | | | | | | | | | | | | | | | | | | | | | | | | | | | | | | | | | | | | | | |  |  |
| **Mode of transportation** | | | | | **Average distance** | | | | | | | **# of days** | | | | **# of trips per month** | | | | | **# of staff who travel** | | | | | **Per diem per person** | | | | | | **Transportation cost/fare per person** | | | | | | | | **Fuel cost** | | | **Notes/comments** | | |  |  |
|  | | | | |  | | | | | | |  | | | |  | | | | |  | | | | |  | | | | | |  | | | | | | | |  | | |  | | |  |  |
|  | | | | |  | | | | | | |  | | | |  | | | | |  | | | | |  | | | | | |  | | | | | | | |  | | |  | | |  |  |
|  | | | | |  | | | | | | |  | | | |  | | | | |  | | | | |  | | | | | |  | | | | | | | |  | | |  | | |  |  |
| III.3 | | Need assessment for MI related vaccine and commodity distribution requirement at this level | | | | | | | | | | | | | | | | | | | | | | | | | | | | | | | | | | | | | | | | | | | |  |  |
| III.3.1 | | Is the current vaccine collection/distribution system adequate to accommodate the additional vaccine distribution needs for future MI interventions? Describe why you say so? | | | | | | | | | | | | | | | | | | | | | | | | | | | | | | | | | |  | | | | | | | | | |  |  |
| III.3.2 | | How would the existing vaccine distribution system and the other MNCH commodity distribution system at this facility need to adapt to accommodate distribution needs for future MI interventions? Describe why you say so? | | | | | | | | | | | | | | | | | | | | | | | | | | | | | | | | | |  | | | | | | | | | |  |  |
| III.3.3 | | What specific elements of vaccine and commodity distribution system would need to be added at this level to introduce MI interventions? Elaborate. *Probe if purchase of new vehicle to aid distribution of vaccine and immunization supplies are deemed necessary.* | | | | | | | | | | | | | | | | | | | | | | | | | | | | | | | | | |  | | | | | | | | | |  |  |
| III.4 | | During vaccination campaigns, how are the vaccine distribution typically handled? If different during vaccination campaigns is different from routine, please describe in detail how it is different.  *(Please consider how this was done during a recent vaccination campaign).* | | | | | | | | | | | | | | | | | | | | | | | | | | | | | | | | | |  | | | | | | | | | |  |  |
| III.5 | | Roles and responsibilities among staff currently working on EPI and MNCH | | | | | | | | | | | | | | | | | | | | | | | | | | | | | | | | | | | | | | | | | | | |  |  |
| III.5.1 | | How would roles and responsibilities work or need to change across EPI and MNCH staff responsible for distribution of vaccines and immunization supplies, if any, for the introduction and delivery of future MI interventions? *(Please note responses separately for different delivery scenarios, as applicable)* | | | | | | | | | | | | | | | | | | | | | | | | | | | | | | | | | |  | | | | | | | | | |  |  |
| III.6 | | What are the biggest opportunities and challenges you foresee around future MI intervention distribution system? | | | | | | | | | | | | | | | | | | | | | | | | | | | | | | | | | |  | | | | | | | | | |  |  |
| III.7 | | Other supply chain and related information | | | | | | | | | | | | | | | | | | | | | | | | | | | | | | | | | | | | | | | | | | | |  |  |
| III.7.1 | | How are logistics management information systems managed for EPI at this level? | | | | | | | | | | | | | | | | | | | | | | | | | | | | | | | | | |  | | | | | | | | | |  |  |
| III.7.2 | | How are logistics management information systems managed for MNCH commodities at this level? | | | | | | | | | | | | | | | | | | | | | | | | | | | | | | | | | |  | | | | | | | | | |  |  |
| III.7.3 | | What data do you currently use to quantify maternal vaccine (e.g., TD) demand? | | | | | | | | | | | | | | | | | | | | | | | | | | | | | | | | | |  | | | | | | | | | |  |  |
| III.7.4 | | What data do you currently use to quantify newborn vaccines (such as BCG birth dose, Hep B birth dose) demand? | | | | | | | | | | | | | | | | | | | | | | | | | | | | | | | | | |  | | | | | | | | | |  |  |
| **IV.1** | | **Waste disposal practices** | | | | | | | | | | | | | | | | | | | | | | | | | | | | | | | | | |  | | | | | | | | | |  |  |
| IV.1.1 | | Where and how is the vaccine waste generated from EPI vaccine administration disposed? Describe. | | | | | | | | | | | | | | | | | | | | | | | | | | | | | | | | | |  | | | | | | | | | |  |  |
| IV.1.2 | | Where and how is the vaccine waste generated from administering pregnant women disposed? Describe. | | | | | | | | | | | | | | | | | | | | | | | | | | | | | | | | | |  | | | | | | | | | |  |  |
| IV.1.3 | | Will it be necessary to establish waste management systems at ANC clinics for future MI vaccines? Describe what additional resources may be needed. | | | | | | | | | | | | | | | | | | | | | | | | | | | | | | | | | |  | | | | | | | | | |  |  |
| **V.1** | | **Human resources** | | | | | | | | | | | | | | | | | | | | | | | | | | | | | | | | | |  | | | | | | | | | |  |  |
| V.1.1 | | How many people work in this health facility? | | | | | | | | | | | | | | | | | | | | | | | | | | | | | | | | | |  | | | | | | | | | |  |  |
| V.1.2 | | How many people at this health facility work in the immunization program including providing immunization services, managing vaccine stocks, or preparing immunization reports? | | | | | | | | | | | | | | | | | | | | | | | | | | | | | | | | | |  | | | | | | | | | |  |  |
| V.1.3 | | On a typical day, when fixed immunization services are provided, how many people work together to provide immunizations services? | | | | | | | | | | | | | | | | | | | | | | | | | | | | | | | | | |  | | | | | | | | | |  |  |
| V.1.4 | | On a typical outreach session, how many people work together to provide immunizations services? | | | | | | | | | | | | | | | | | | | | | | | | | | | | | | | | | |  | | | | | | | | | |  |  |
| V.1.5 | | On a typical campaign, how many people work together to provide immunizations services? | | | | | | | | | | | | | | | | | | | | | | | | | | | | | | | | | |  | | | | | | | | | |  |  |
| V.1.6 | | How many people at this health facility work in the MNCH/ANC program including providing antenatal care services? | | | | | | | | | | | | | | | | | | | | | | | | | | | | | | | | | |  | | | | | | | | | |  |  |
| V.1.7 | | On a typical day, when ANC services are provided, how many people work together to provide ANC services? | | | | | | | | | | | | | | | | | | | | | | | | | | | | | | | | | |  | | | | | | | | | |  |  |
| V.1.8 | | On a typical outreach session, how many people work together to provide ANC services? | | | | | | | | | | | | | | | | | | | | | | | | | | | | | | | | | |  | | | | | | | | | |  |  |
| V.1.9 | | How many health volunteers are attached to this health facility specifically for EPI? | | | | | | | | | | | | | | | | | | | | | | | | | | | | | | | | | |  | | | | | | | | | |  |  |
| V.1.10 | | How many health volunteers are attached to this health facility specifically for MNCH program? | | | | | | | | | | | | | | | | | | | | | | | | | | | | | | | | | |  | | | | | | | | | |  |  |
